# Selections

**Published:** 1817-03

**Authors:** 


### PART III.

### SELECTIONS.

—— *Factorum est copia nobis.*

### FOREIGN JOURNALS.

PHYSIOLOGY.—*Memoir on the First Inspiration of the new-born Child.**

( Continued from page 159. )

III. It may again be objected, that my explanation of the phænomenon of the first inspiration applies exclusively to natural labour, and not to those cases in which the head of the fœtus, after being some time detained in the pelvis, is last expelled. Here, it will be urged, however powerful be the compression of the thorax, and consequent contraction of the diaphragm, they will avail nothing; for inspiration cannot take place so long as the head remains in the pelvis: and, on the other hand, when this part of the child is at length expelled, it may be presumed that the excitement of the diaphragm has already ceased. Yet children, thus delivered, respire as soon as they come to light, provided the delivery be effected without great difficulty, and by the mere efforts of nature. Hence it becomes necessary to admit, either that the diaphragm is subjected to repeated contractions during the whole time of retention of the head in the pelvis, or that the muscle is excitable by other causes than those hitherto specified. I indeed admit, that the diaphragm may be excited by other means as well as by compression of the ribs. In foot delivery, the very weight of the liver operates as a stimulus upon this exquisitely irritable muscle. A similar effect will be produced on the diaphragm, when, in extracting the fœtus from the womb by the Cæsarean operation, it is held in a vertical position: and who knows but that, in the case of children still-born in consequence of turning, and upon whom no luxation of the cervical vertebræ, or other organic lesion, is discovered, the event happens from the trunk of the fœtus not being in the disposition which I consider essential for the excitement of the diaphragm and intercostals. Such cases then, it is obvious, far from being hostile, are favourable to my opinion. Again, I admit that the change of temperature to which the fœtus, on its

* Bulletin de la Société Médicale d'Emulation, No. IV. Avril, 1816.

birth, is exposed, the stimulus of cold air, and of the different motions to which it is subjected, may excite, in the muscular system, contractions subsequently propagated, by a kind of association, to the diaphragm. Thus I explain the facts observed by Bayle* and Vesalius;† who, on removing the impregnated uterus from a living animal, and incising the membrane of the ovum, saw the young ones immediately respire. But we know that, in such an operation, the womb must necessarily be much disturbed, and the young animals compressed and shaken, as is proved by their agitation, previously to the incision of the containing organ. Nor is it surprising, that the general commotion which they experience, should be reflected to the inspiratory muscles. Once more, the means of exciting the contractions of the diaphragm are various: nevertheless, there is one only which we may term regular, and which nature employs in preference to others. An analogy, in my opinion, exists between the fœtus just born and an individual imperfectly asphyxiated; and I compare the different means of resuscitating the individual, such as friction, irritation of the membrane of the nostrils and rectum, and electricity, to the manœuvres executed on the infant or other young animal, in the view of acting sympathetically on the respiratory muscles. But there is another process for the recovery of asphyxiated infants, equally useful; and which, indicated by the celebrated Albinus,‡ still bears his name. This consists in cautious compression of the thorax, particularly at its base, and then suffering the ribs to rebound. Thus the diaphragm and intercostals are most directly acted upon; and this, in my opinion, is the very mean employed by Nature to put in action the fœtal organs of respiration. For half a century, this process has been taught in the schools,§ universally known, and practised by accoucheurs: and no one doubts that, in thus acting, he imitates Nature.

IV. A last objection may be urged against the insignificance of the means by which respiration is excited. Is it probable that Nature would confide to an act so inconsiderable as compression of the ribs, a function essential to all organized beings, and, consequently, of such high importance? But, even forgetting that Nature, in her operations, frequently produces great results by trivial means, we may affirm, that the constriction suffered by the fœtus in birth

* Experiences Physiolog. Mechan. p. 41.

† Bœrhaave, Prælect. in Propr. Institut. t. v. part 2, p. 383. "Vesalius Bononiæ, Patavii et Pisis experimenta fecit (p. 824, ed. 1555). Exemit ex vivo bruto, verbi gratiâ ex porcâ, uterum unâ cum fœtubus, integrum in mensâ anatomicâ explicavit, ita vidit animalcula miris modis se movere et nixus edere quos nemo a tantillis bestiolis expectasset: dissecabat uterum et amnion, continuò respirabant omnia."

‡ Haller, Element. Physiolog. t. iii. p. 225.

§ Storcheus, Kinderkrankheiten, 1. B. p. 79, 80. Roederer, Observat. Med. Satur. de Suffocatis. Opusc. Med. t. i. p. 2, 306. Frank, System der Medicin. Polizey, B. 5, S. 36.

is become in our view a phænomenon of little consequence, only from want of due reflection upon it. Far then from regarding it as a trivial circumstance, I descry in it a law of Nature, by which the fœtus of man and quadrupeds is expelled with difficulty, in order that the grand function, distinguishing the born from the unborn child, may be established. Hence women bring forth with difficulty. Had it been the design of Nature to facilitate the process, another mode of delivery might have been instituted, exempt from these pains and difficulties. But what had then happened? The fœtus would have been expelled, such as it had previously been, lethargic, without respiration or motion, and, as it were, asphyxiated. How then does experience here accord with theory: it proves that, in deliveries unduly rapid, infants are expelled in a state of apparent death; which, unless assistance be given, becomes real. Is it not partly from this reason that juridical physicians admit the innocence of a woman accused of infanticide, when the undue rapidity of her delivery has been ascertained?

The effects of the compression suffered by the fœtus in birth are not, however, limited to the thorax; they equally affect the cranium. This part of the child is longest exposed to the compressive forces, which even cause the margins of the cranial bones to cross each other; but the head, on emerging from the inferior pelvic aperture, passes from a state of the utmost constriction to that of perfect freedom. This sudden change of form necessarily influences the brain itself, to which it must communicate a decided shock. Now I maintain, that Nature has not made this arrangement without design. The fœtus, which, while enclosed in the womb, leads a vegetative life, is, on birth, to be placed in relation with surrounding objects, and to exercise at once the sensual organs, and those of voice and locomotion, which are all in immediate dependence on the brain; like machines, which, to be put in motion, require but an impulse from the hand of the artist. I imagine, that the fœtus must commence its life of relation by a shock of the cerebral organ, which again excites the nervous system, and the organs submitted to its empire. Bichat* has proved, that the brain of adult man is in constant mechanical excitement, produced by the action of the arteries situated at its base, and that this excitement contributes powerfully to support the functions of the organ. That which I admit in the fœtus, must act much more powerfully than the other. It certainly operates in an inverse direction, that is, from the summit of the brain to the base; but the influence on the organ must be the same. It is, doubtless, for this purpose, that Nature has placed the brain in a compressible and elastic cavity, and formed the cranium of several pieces, united by membrane, and hence favouring the reduction of its diameters: a disposition refused to birds, which differ in their mode of birth from man and quadrupeds, and hence possess no costal cartilages. The

* Expériences sur la Vie et la [illegible], p. 201, 207.

head of the human fœtus might have been formed of a single piece, and the osseous portion of the ribs prolonged to the sternum, as in birds. But then compression of the cranium and thorax would not have been so easy, and the instantaneous restoration of these parts to their original state would not have produced the necessary shock. Doubtless, from the compressibility of the cranium and thorax, the fœtus passes more readily through the pelvis: but, again, why is the infant destined to pass through a space so contracted? It is, I repeat, because Nature has imposed a law by which all the females of the human species, and of quadrupeds, bring forth with pain and difficulty, in order that their young may receive the excitement necessary for the exercise of the functions to be established after birth; so that their entrance upon the world, and commencement of a new life, are signalized by a kind of revolution in their organism.

Such are my ideas on the first inspiration of the fœtus; which I feel gratified in finding conformable to those of two celebrated physicians in the schools of Germany, Hamberger and Roederer. The first expresses himself in these terms: "Transitus fœtus per genitalia muliebria fieri nequit, quin ejus pectus atque abdomen insigniter constringantur. Si igitur caput in partu ostendit, omnes costæ fœtûs deorsùm premuntur, et sic musculi intercostales externi extenduntur atque ad contractionem irritantur. Simili modo ex abdominis durante partu constrictione, diaphragma sursum pellitur et ad contractionem irritatur. Primo ergò momento, quo partu peracto extensio diaphragmatis atque externorum intercostalium musculorum cessat, hi tamquam irritati sese contrahunt et pectus dilatant."* And Roederer, in alluding to an hypothesis attributed to Boerhaave, thus declares himself: "Lubens sane istam hypothesin (Boërhaavii) amplecti deferemus qùa in ipso partu tota corpusculi superficies ob arctissimam compressionem irritatur eo quidem consilio ut natus infans omnes musculos agitet; quod si hoc fit, non potest non thoracis musculos et abdominis simul ita movere ut thorace dilatato pulmones expandantur."†

### PATHOLOGY.—*Memoir on Inflammation of the Hernial Sac.*‡

( Concluded from p. 167. )

*Second Case.* Koepfer, a tailor, aged 70, had been affected from his earlier years with inguinal hernia of the left side. Notwithstanding the use of an elastic truss, the hernia frequently came down, and produced severe symptoms; but its reduction was invariably effected. Long an inmate of *l'Hospice de Bicetre*, in con-

---

* Physiologia Medica, § 1595.

† Observat. Medic. Satur. de Suffocatis. Opusc. Med. t. i. p. 2, page 306.

‡ See Bulletin de l'Athénée de Médecine de Paris. Juin, 1816.

sequence of humoral asthma, he would no more submit to the inconvenience of wearing a truss, although he got up and walked about the ward.

In June, 1811, the hernia was reproduced by the efforts of the cough, and became strangulated; and taxis, aided by the ordinary means, was long employed before it could be reduced. Some graduated compresses were then applied upon the ring, and retained by a bandage. Yet uneasiness came on, succeeded by pain beneath the pad; respecting which, the patient, a few days afterwards, consulted me. I found at the ring a tumour, as large as a pullet's egg, somewhat flattened, pyriform, resisting, and almost indolent, but painful on pressure. It was moveable in all directions, except towards the ring, which it adhered to by its summit, and even seemed to penetrate. The ring appeared much dilated. All attempts at reduction were fruitless; but, as no symptoms of strangulation existed, I left the tumour to itself. I at first thought that a portion of omentum, unreduced and swollen from compression of the bandage, had given rise to it.

But incited by the preceding fact, which had just fallen under my observation, I paid more attention to this new case. The tumour neither increased nor diminished in the various postures assumed by the patient, notwithstanding the violence of the cough and other efforts. Although resistent, it was depressed by strong pressure, and yielded a sense of crepitation; but, on removal of the pressure, it resumed the original state. Some analogy was then thought to exist between this tumour and that which makes the subject of the first case. I announced that it was formed by alteration of the hernial sac, consisting in tumefaction and chronic inflammation of its parietes; and my opinion was confirmed by dissection on the 20th of August following, the patient having been destroyed by his asthma. In this interval of two months, the tumour had scarcely increased in volume, and the integuments covering it were sound.

Dissection.—*Abdomen.* All the viscera were in their natural site and relations, except that the small intestines were directed a little to the left. The peritonæum dipped into the left abdominal ring, in the shape of a funnel. A contraction, situated there, was destroyed with little effort by the finger, which thus penetrated into the interior of the tumour. This, like the former, contained a puriform, viscous, whitish albuminous matter. Its internal surface was smooth and shining, somewhat translucid, less red than that of the first, and not at all corroded. Its external surface was white, and confounded with the surrounding cellular structure. The parietes were also white, two or three lines thick, very dense, and exhibiting the appearance of a scirrhous degeneration, of the first degree. The layers, which I removed from it, seemed to adhere more strongly as I approached the internal surface. They were probably formed by cellular structure, which shared the morbid alteration.

*Remarks.* Here the tumefaction of the sac evidently succeeded the reduction of the hernia; for immediately after, I perceived no trace of it. The irritation excited by the presence of the displaced parts, and the reiterated attempts at reduction, aggravated by the compression of the bandage, seem to have acted as the exciting cause. The fluid contained in the tumour was less altered than in the first case. Its internal surface was less red, and not corroded; doubtless, because the inflammation, which was active in the former case, preserved, in the latter, a chronic character. If, by any means, abdominal inflammation, or volvulus, had been complicated with this tumour, a surgeon, not apprized of the case, would have been readily imposed upon; and, presuming that the tumour and attendant symptoms were attributable to strangulated hernia, have had recourse to an useless operation, as in the first case. This error is the more likely to be committed, inasmuch as frequently in strangulations, the pain is referred by the patient, not to the tumour, but to some part of the abdomen.

*Third Case.* M——, aged 55, free from all complaint, except what was caused by the descent and strangulation of an inguinal hernia, to which he had been subject from his youth, felt in June, 1811, an uneasiness, at first slight, under the pad of his truss. Its increase led him to investigate the cause. He found at the abdominal ring a tumour of the size of a large nut, somewhat flattened, elastic, and resisting. In the idea that it was hernia, he made useless attempts to reduce it. But, at last, finding no other inconvenience, and the digestive powers continuing undisturbed, he replaced his truss, and resumed his original employment. Nothing particular occurred for six weeks. But, at the end of that time, the tumour became painful and increased in size, probably from the symptomatic tumescence of the surrounding cellular structure, the accumulation of fluid in the cavity of the tumour, and congestion of its parietes. The truss, no longer tolerable, was removed. The tumour was considered as formed by the inflammation of a lymphatic gland. Emollient poultices were applied, and fluctuation, though deep-seated, became evident, through the thick and indurated parietes of the tumour. Presently, from several orifices spontaneously formed, was discharged about an ounce of pus, containing albuminous flakes. I then first visited the patient. The situation of the tumour confounding itself with the abdominal ring, the antecedent circumstances, and its previous and present characters, left no doubt as to its nature. It evidently consisted in inflammation, at first latent, and subsequently acute, of the parietes of the hernial sac. Although completely empty, it retained the original size; because its elastic and resisting parietes could not collapse. It thus exhibited a gaping cavity, containing pus somewhat viscous on the first dressings. The resistance, and especially the elasticity of its parietes, caused them to rebound on removal of pressure. The cavity was prolonged a little into the abdominal ring. The continual application of emollient poultices, of suppuratives and detersive and irritating injections, effected, in two months, the almost complete subsidence of the parietes of the tumour, and the entire cicatrization of its orifices, till then fistulous. During all this period, the hernia never reappeared, although the patient walked much after the abscess had formed, and the violence of the inflammation was abated. His truss was completely re-constructed. Having lost sight of this patient, I know not whether the hernia has recurred. Would not the occlusion of the abdominal ring by the remnant of the parietes of the sac, and the solidity of the cicatrix, have sufficed to obviate a fresh displacement, and rendered unnecessary the employment of a truss?

*General Reflections on the three Cases.*—By what parts could these peculiar tumours have been formed, similar as they, in all respects, were, especially in the two first cases, except by the hernial sac? In fact, in all abdominal and particularly inguinal herniæ, the sac contracting adhesions and intimate relations with the inguinal region, can no longer be reduced with the displaced parts. It is this which, for the most part, disposes to the progressive frequency of displacement; and must have existed in the subjects of my observations. On the other hand, I found no other trace of sac than the tumours in question, which occupied exactly its situation, and nearly affected its form and aspect. Indeed, 1st. The tumours issued like a sac from the abdominal ring, and seemed to originate by a neck of peritonæum which, funnel-like, dipped in behind the ring. 2dly. Like it, they had a pyriform figure, and immediately lay on the vessels of the spermatic cord. 3dly. Like it, they had a cavity, the internal membrane of which precisely resembled a serous membrane. Lastly, The nature of the fluid of the tumours, rather puriform than purulent, containing albuminous flocculi; and, in short, resembling the product of inflammation of serous membranes, proves that they were formed by the morbid hernial sac.

In the third case, I had not, as in the other two, the advantages of anatomical inspection. Yet, on attentively examining the analogy which exists between the causes, symptoms, and characters, the exterior aspect, the physical qualities of the fluid discharged from the spontaneous apertures, and the relations of this tumour with the two former, no doubt can exist as to the identity of their nature and situation.

The seat of the affection thus proved, let us try to ascertain its cause. This, as I have before particularly observed, seems to consist in tumefaction of the hernial sac, with inflammation, acute or chronic, whether as a cause or consequence of, or complication with, such congestion. Thus, then, either latent inflammation has induced the successive congestion of the parietes of the sac; or acute inflammation has suddenly determined it; or, lastly, the congestion has been, for a time, established without evident inflammation, the latter having only supervened from the operation of the causes indicated.

In either case, the morbid alteration of the sac necessarily induces a change in the product of secretion; and the congestion, spreading to the adjacent cellular structure, thus contributes to the thickening of the parietes and augmentation of the volume of the tumour.

From the cases thus described, and the reflections which they naturally excite, there can be no doubt as to the occurrence of alteration of the hernial sac, consisting in tumefaction and inflammation, acute or chronic, of its parietes, with change in the product of exhalation, as existing without actual displacement, and thus constituting an essential disease.

(This Memoir will be terminated in our next Number. EDIT.)

---

### *Narrative of an uncommon Case of Catalepsy.**

F. J. Busch, a soldier in the 4th regiment of Dragoons, born in the department of the Lower Rhine, aged 28, of a feeble constitution and lymphatic temperament, long subject to moral affections and every kind of difficulty and privation, entered, on September the 23d, 1815, the military hospital of Montaigu, in the following state:—There were general stupor and dejection; exercise of all the senses suspended; habitual somnolence; symptoms of catalepsy commencing; tension of the abdominal muscles; sensibility of the epigastrium and globe of the eye, which the constant blinking of the eye-lids sometimes exposed to the action of light; irregular motions of the hands and fingers, which might be confounded with carphologia; no appetite. Life seemed to have forsaken the external organs, in order to concentrate itself in the feeble functions of circulation and respiration. On my first visit, I was undecided as to the character of the disease, and the diagnosis was the more difficult, as I knew nothing of the circumstances which had preceded or determined its existence. I only was informed that he had been discharged from *l'Hopital St. Louis,* where he had resided from the 22d of August; that, during his stay there, he had been constantly sullen and dejected, and that bitters had been administered. However it were, something must be immediately done; and I thought that the character of weakness and insensibility was sufficient to authorize the employment of the most powerful remedies. At first, I had recourse to vomiting and tonic drinks. I prescribed three grains of tartrite of antimony in an ounce of oxymel of squills, and an infusion of the flowers of arnica, and canella wine. These seemed to arouse the patient and excite extraordinary motions of the thoracic limbs. He several times raised his hand to his head, as if to indicate the seat of the disease, and sighed deeply: but this was of short duration. He soon relapsed into a comatose state, and the symptoms of catalepsy were de-

---

* Observation d'un Cas Rare de Catalepsie; par M. Boussenard, D. M. &c. Bulletin de l'Athénée de Médecine de Paris, Mai, 1816.

veloped with violence. The muscles, perfectly supple, obeyed every impression, and allowed the limbs to assume and long preserve the most difficult attitudes. I found him, one day, seated on his bed, his hands united in the form of a desk, with the palmar surface upwards, and a book upon them. This position he preserved about two hours, which was the longest term noted by me during the whole course of the disease. The exhausted muscles then yielded to the weight of the limbs, which resumed their natural attitude: I, at that time, felt convinced that the catalepsy was dependent on an organic lesion of the brain or its membranes; and hence my chief attention was directed to it. The head was covered with a blister; sinapisms were applied to the legs; and to the ptisans, before prescribed, were united preparations of ammonia, the ethereal wines; and frictions, with tincture of cantharides. Panada constituted the base of the dietetic plan. Two nurses were appointed to attend the patient, and Dr. Saclandière had the kindness to superintend the execution of the prescriptions. A month passed without any sign of amendment. At this period, a cataplasm of ice, applied upon the head, produced the following effects:—The patient was violently excited; screamed; made great motions, and evinced a wish to articulate. The face was highly flushed; the pulse strong and accelerated; respiration quick and laborious. Profuse sweats announced a salutary crisis, but depression succeeded the arduous effort, and the patient relapsed into his ordinary state.

I wished to try electricity; but no machine could be procured. I, therefore, continued the frictions generally; and employed alternately, flagellation, urtication, sternutatories, blisters, *moxa* on the course of the lesser sympathetic nerve and vertebral column, the sound of harsh music; and, internally, phosphorus and extract of nux vomica. But all were useless: Each mode of irritation had but a momentary effect, invariably succeeded by extreme atony. Persuaded that the disease would be of long duration, and fearing to exhaust the sensibility by a continued use of exciting remedies, I determined to leave it principally to the resources of nature; and hence reduced the therapeutic plan to tonic potions and a generous diet. Good wine, panada with the yolk of an egg, biscuit, and sugar, were allowed; and to these were added cleanliness, pure air, and oxygenated fumigations. Of all the secretions, it should be observed, that of the urinary organs was alone copious. The constipation was obstinate, and resisted the repeated employment of injections and calomel. The patient voided only every three or four days, with the utmost difficulty, blackish indurated fæces, resembling in consistence, the dung of certain animals.* The scanty perspiration

---

* We are surprized that this circumstance did not strongly arrest the attention of Dr. Boussenard, and constitute, in his view, a sufficient indication for the full trial of a course of purgative medicines. Edit.

had a strongly ammoniacal odour, approaching that of beasts of prey.

All these phænomena were observed by several physicians of the capital, who examined the patient and favoured me with their opinions respecting him. They were persauded that the disease would terminate fatally, and that it would be useless to try any other treatment. In fact, five months had already elapsed; and extreme weakness, emaciation, and refusal of food, announced a speedy close. The scurvy, which prevailed among several patients of the ward, was communicated to him, and made rapid progress. Diarrhœa succeeded to constipation. The patient, apprized by his feelings of a motion to go to stool, placed his hand so as to receive the discharge of fæces. Their offensive odour with that issuing from his mouth and whole body, obliged me to place him in a separate ward under the care of an attendant. The scorbutic symptoms increased daily in violence; large ecchymoses occupied several parts of the body; the smell of the mouth became intolerable; the fæces, often mixed with blood, evinced the last degree of scorbutic virulence: all, in short, bespoke a speedy dissolution.

Yet some changes, which might be regarded as preparatory to the grand crisis of the disease, had been, for a time, observed: there was no longer any tension of the abdomen; nor was the epigastrium now the only centre of sensibility which, universally diffused, developed itself on the least touch. The cutaneous system was no longer inactive. The patient sometimes opened his eyes, and turned them to surrounding objects. His attendant one day surprized him raising the vessel, which contained the ptisan to his mouth. The cold, to which he had previously been insensible, inconvenienced him much on removal of his covering, or excited groaning and complaints.

But all these signs made no impression on me. Occupied with the scurvy, I still delivered the most unfavourable prognosis. And great was my astonishment, when, on my morning visit, of May 28th, 1816, the patient entreated, in German, to have a priest, some bread, and wine. The two latter were immediately given him; and he swallowed them with avidity, so that I had no doubt of his speedy cure. In fact, thence forward, his symptoms gradually decreased; and extreme debility is the only one now remaining. All the signs of catalepsy have disappeared: the digestive organs are recovered: the patient gets up and walks about; but the intellectual functions are yet little restored: he has no recollection of what passed during his illness. He remembers that, from his earliest years, he was constantly abused by his relatives; that he was most reluctantly dragged by the conscription to the army; that, as a private of the 7th regiment of the line, he was in the campaign of Russia; that, on his return, he was enrolled in the 4th Regt. of Dragoons; that, from his inaptitude to mount his horse, he was frequently exposed to ill-treatment from the quarter-master, who, one day, struck him on the head so violently, that he was carried senselesss to the hospital; whence, from the events of the war, he was sent to the *L'hopital Saint-Louis*, of Paris.

Such is the sketch of a disease, scarcely paralleled, either in the duration or violence of its symptoms, by any example which writers on catalepsy have yet recorded. Boërhaave is mistaken in regarding this affection as fatal. I have once before met with a case of it which invalidates his opinion.

The disease commonly originates in moral affections; but these, I think, in the present case, can only be considered as predisponent causes; and the blows on the head as in reality the exciting cause: for, from the period of their infliction the symptoms first became manifest. I am ignorant how the crisis was effected; uncertain whether it may be correctly attributed to the bloody stools which were voided some days previously to convalescence.

---

### *Epilepsy cured in consequence of a scirrhous Affection of the Testicle.**

A man, aged 30, had been subject, from his infancy, to attacks of epilepsy, originally attributed to terror. He was, in his youth, of an ungovernable temper, and several times got into prison. The epileptic paroxysm recurred every eight or fifteen days, and was characterized by sudden invasion, convulsions, and utter insensibility during several minutes. From his eighteenth to his twenty-fifth year, the paroxysms were less frequent but stronger. About this period, the patient, several times, contracted simple gonorrhœa, which seemed to retard the fits. In 1812, he experienced an ophthalmia which resisted all the ordinary remedies, and was followed by enlargement of the left testicle. For this, the antisyphilitic treatment was instituted; which in conjunction with a seton in the neck removed the ophthalmia, but made no impression on the testicle. In 1813, the organ had acquired the volume of an egg. I tried mercurial frictions on the internal part of the left leg and thigh, and even on the scrotum. But the disease progressively increased; and the epileptic paroxysms sensibly diminished in frequency and force. A few fits only occurred in 1814. In 1815, the testicle had attained the size of a child's head. It was not painful; and scarcely inconvenient but from its volume and weight; though kept up by a suspensory bandage. On the 3d of November, 1815, I removed the patient to the sur-

---

* Epilepsie guérie a la suite d'une dégénérescence squirrheuse du Testicule gauche. Par M. Hébréard Bulletin de l'Athénée, &c. Septembre, 1816. We much doubt whether this were a really scirrhous affection. The writer, himself, observes in a note, that "the testicle was in a state of fatty degeneration, pervaded by small cysts of purulent matter, and without any trace of the seminal tubes." Edit.

gical ward, where the testicle was extirpated by M. Dumont. At the close of December, the wound was completely cicatrized. For seven months before the operation, the patient had no return of the epileptic fits; and not only has he since been free from them; but his character, previously irritable and impetuous, seems to be much softened and subdued. He has also gained flesh since the operation.

---

